# Treatment of dyslipidemia in patients with type 2 diabetes

**DOI:** 10.1186/1476-511X-9-144

**Published:** 2010-12-20

**Authors:** Krishnaswami Vijayaraghavan

**Affiliations:** 1 Cardiovascular Division, Scottsdale Healthcare Research Institute, Scottsdale, AZ, USA

## Abstract

Type 2 diabetes is associated with significant cardiovascular morbidity and mortality. Although low-density lipoprotein cholesterol levels may be normal in patients with type 2 diabetes, insulin resistance drives a number of changes in lipid metabolism and lipoprotein composition that render low-density lipoprotein cholesterol and other lipoproteins more pathogenic than species found in patients without type 2 diabetes. Dyslipidemia, which affects almost 50% of patients with type 2 diabetes, is a cardiovascular risk factor characterized by elevated triglyceride levels, low high-density lipoprotein cholesterol levels, and a preponderance of small, dense, low-density lipoprotein particles. Early, aggressive pharmacological management is advocated to reduce low-density lipoprotein cholesterol levels, regardless of baseline levels. A number of lipid-lowering agents, including statins, fibrates, niacin, and bile acid sequestrants, are available to target normalization of the entire lipid profile. Despite use of combination and high-dose lipid-lowering agents, many patients with type 2 diabetes do not achieve lipid targets. This review outlines the characteristics and prevalence of dyslipidemia in patients with type 2 diabetes and discusses strategies that may reduce the risk of cardiovascular disease in this population.

## Introduction

Type 2 diabetes affects approximately 24 million individuals in the United States [1] and is associated with significant morbidity and mortality due to cardiovascular complications [2]. The incidence of cardiovascular disease (CVD) is more common in patients with type 2 diabetes than in the general population [3]. Dyslipidemia, an established risk factor for CVD, is strikingly common in patients with type 2 diabetes, affecting almost 50% of this population [4]. In addition to hyperglycemia and hypertension, dyslipidemia is a modifiable CVD risk factor that remains largely uncontrolled in patients with type 2 diabetes [4].

Hyperglycemia increases the risk of microvascular complications [5], while dyslipidemia is a major risk factor for macrovascular complications in patients with type 2 diabetes [6,7]. Elevated low-density lipoprotein cholesterol (LDL-C) is a major risk factor for CVD [6]. As such, management of LDL-C is the primary goal of therapy for diabetic dyslipidemia [8-10]. Furthermore, type 2 diabetes increases the risk of CVD mortality independent of LDL-C levels, adding to the greater overall cardiovascular risk in this population [11]. Therefore, aggressive lipid treatment goals have been recommended for patients with type 2 diabetes (Table 1) [8-10,12]. As the prevalence of type 2 diabetes increases in the United States, prevention of CVD is becoming an increasingly urgent public health concern, requiring aggressive management of the entire lipid profile [8]. This review outlines the characteristics and prevalence of dyslipidemia in patients with type 2 diabetes and discusses strategies that may reduce the risk of CVD in this population.

**Table 1:** Low-density Lipoprotein Cholesterol (LDL-C) and Non-HDL-C Goals for Patients in Different CVD Risk Categories from the Adult Treatment Panel III of the National Cholesterol Education Program [12,30]

Risk Category	Goals (mg/dL)	
	
	Primary target: LDL-C	Secondary target: Non-HDL-C^‡^
CVD + T2DM (CVD risk equivalent)*	< 70	< 100

CVD or T2DM^†^	< 100	< 130

≥ 2 risk factors (not CVD risk equivalents)	< 130	< 160

0-1 risk factor (not a CVD risk equivalent)	< 160	< 190

CVD, cardiovascular disease; HDL-C, high-density lipoprotein cholesterol; T2DM, type 2 diabetes mellitus; non-HDL-C = LDL-C + very-low-density lipoprotein cholesterol (VLDL-C) or total cholesterol - HDL-C.

*In addition to reduction of LDL-C to a goal of < 70 mg/dL as an option in very high-risk patients with overt CVD (level of evidence B), additional American Diabetes Association (ADA) recommendations are the reduction of LDL-C by 30%-40% in all patients with diabetes and overt CVD, regardless of baseline LDL-C levels (level of evidence A); lower triglycerides to < 150 mg/dL and raise HDL-C to > 40 mg/dL, with the option of > 50 mg/dL in women (level of evidence C) [10].

^†^In addition to the reduction of LDL-C to < 100 mg/dL as the primary therapeutic goal (level of evidence A), the ADA recommends that LDL-C be reduced by 30%-40% in all patients with diabetes > 40 years of age without overt CVD, regardless of baseline LDL-C levels (level of evidence C) [10].

‡Non-HDL-C is a secondary target of therapy in patients with high serum triglycerides (≥ 200 mg/dL) [30].

## Characteristics and mechanisms of lipoprotein abnormalities in type 2 diabetes

**Figure 1:**
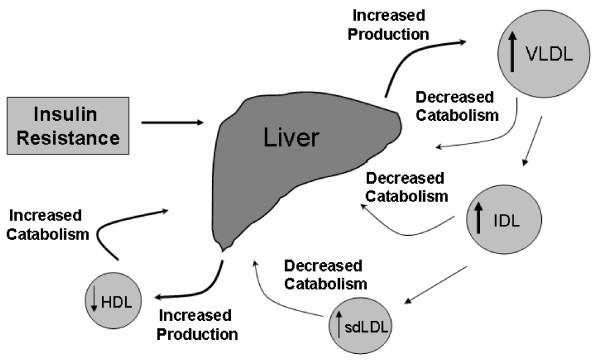
**Atherogenic dyslipidemia and changes in lipoprotein metabolism associated with type 2 diabetes mellitus **[51]. Insulin resistance is associated with enhanced production of very-low-density lipoprotein (VLDL); a reduction in the catabolic rate of intermediate-density lipoprotein (IDL) and small, dense low-density lipoprotein (sdLDL); increased production of high-density lipoprotein (HDL) outweighed by increased catabolism. Adapted with permission from Adiels et al, Overproduction of very low-density lipoproteins is the hallmark of the dyslipidemia in the metabolic syndrome. *Arterioscler Thromb Vasc Biol *28(7): 1225-1236 (2008) [14].

The hallmarks of type 2 diabetes are hyperglycemia, insulin resistance, and insulin deficiency, and it is increasingly recognized that insulin resistance contributes to the characteristic dyslipidemia associated with type 2 diabetes [13]. Disturbance of lipid metabolism appears to be an early event in the development of type 2 diabetes, potentially preceding the disease by several years [14]. In addition, the different components of diabetic dyslipidemia (plasma lipid and lipoprotein abnormalities) are believed to be metabolically linked [13,15]. The dyslipidemia associated with insulin resistance (also referred to as atherogenic dyslipidemia) is characterized by moderately increased triglyceride (TG) levels carried in very-low-density lipoprotein (VLDL) particles, reduced high-density lipoprotein cholesterol (HDL-C) levels carried in small HDL particles, and LDL-C levels that do not differ substantially from those of individuals without type 2 diabetes (Figure 1) [13,15-17]. In addition, TG-rich lipoproteins (after eating), remnant lipoproteins, apolipoprotein B 100 (ApoB), and small, dense HDL particles have also been shown to be increased in patients with type 2 diabetes [18]. In patients with type 2 diabetes, LDL particles are small and dense, carrying less cholesterol per particle; therefore, at any given LDL-C concentration, there are more LDL particles present in an individual with type 2 diabetes relative to an individual without the disease, which may make the LDL-C level a misleading measure of risk in patients with type 2 diabetes [8].

The association between hyperglycemia and microvascular complications in type 2 diabetes is unequivocal [5,19]. However, dyslipidemia may correlate more directly with cardiovascular complications [2], and mechanistic evidence is emerging regarding the greater lipocentric versus glucocentric nature of CVD risk in patients with type 2 diabetes [7,18]. Insulin resistance is central to the pathogenesis of type 2 diabetes and contributes to dyslipidemia [17]. Insulin resistance is associated with increased levels of serum insulin and depletion of β-cells and results in impaired regulation of circulating lipoprotein and glucose levels [7,13]. Data suggest impairment in the ability of insulin to suppress hepatic production of large TG-rich VLDL (VLDL-TGs) in patients with type 2 diabetes results in a subsequent elevation in plasma TG levels (Figure 1) [13-15].

Impaired insulin action at the level of the adipocyte is believed to result in defective suppression of intracellular hydrolysis of TGs with the release of nonesterified (free) fatty acids (NEFAs) into the circulation [17]. The increased influx of NEFAs to the liver promotes TG synthesis and the assembly and secretion of large VLDL; this results in elevated plasma VLDL levels (hypertriglyceridemia) and postprandial hyperlipidemia that is compounded by impaired lipoprotein lipase activity - the latter regarded as independently associated with coronary artery disease. Hypertriglyceridemia can trigger thrombogenic alterations in the coagulation system. In addition, elevated VLDL-TGs reduce levels of cardioprotective HDL-C as TGs are transferred when these particles collide. The reduction in HDL-C levels is accompanied by a reduction in antioxidant and anti-atherogenic activities. Of note, VLDL-enhanced TG enrichment of HDL-C and LDL-C by exchange of TG for cholesterol is followed by hepatic lipase-mediated hydrolysis of the TG portion, resulting in small, relatively cholesterol-poor HDL (which are subsequently catabolized and cleared from the circulation) and LDL particles [18]. When LDL particles become small and dense, they are more prone to oxidation and more readily adhere to and subsequently invade the arterial wall, contributing to atherosclerosis; small, dense LDL particles are therefore regarded as more atherogenic than their larger, more buoyant precursor [17].

Cholesterol ester transfer protein (CETP) is also known to play a pathological role in diabetic dyslipidemia, particularly in the reduction of HDL-C levels due to increased catabolism [20]. This finding has lead to the speculation that CETP inhibition may increase HDL-C levels, and several CETP inhibitors are in development. Failure of the first of these, torcetrapib, due to increased incidence of adverse cardiovascular events, has led to concerns about the utility of CETP as a therapeutic target. However, since increases in blood pressure (BP) and plasma aldosterone levels were reported with torcetrapib, it is possible that off-target effects were responsible for the adverse outcomes rather than CETP inhibition itself [21]. BP and plasma aldosterone increases have not been reported with anacetrapib - the second CETP inhibitor in development [22,23].

Activation of the renin-angiotensin-aldosterone system (RAAS) can interfere with insulin signaling as well, promoting and exacerbating insulin resistance in patients with type 2 diabetes. RAAS activation increases oxidative stress, decreases nitric oxide production, and activates protein kinase signaling pathways, leading to excess generation of angiotensin II, endothelial damage, and vascular complications in patients with type 2 diabetes, resulting in an increased risk of CVD [24,25]. Inhibition of RAAS with an angiotensin-converting enzyme inhibitor reduces the risk of death from cardiovascular causes in patients with type 2 diabetes [24,26].

## The prevalence of dyslipidemia in association with type 2 diabetes

**Figure 2:**
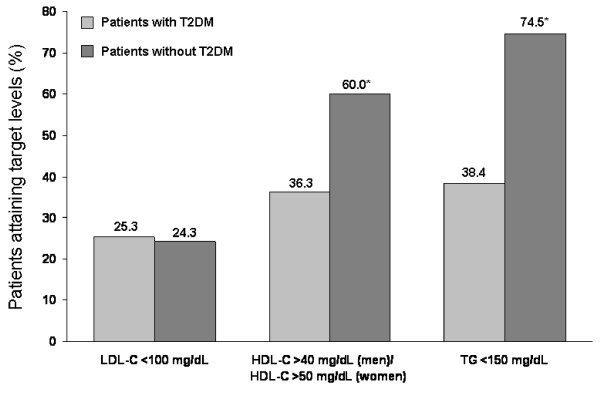
**Prevalence of target low-density lipoprotein cholesterol (LDL-C), high-density lipoprotein cholesterol (HDL-C), and triglyceride (TG) levels for patients with and without type 2 diabetes mellitus (T2DM): National Health and Nutrition Examination Survey 1999-2000**. *P <0.001 vs patients with T2DM. Reprinted from *Diabetes Res Clin Pract*, 70(3), Jacobs MJ, et al, 263-269, Copyright 2005, with permission from Elsevier [27].

**Figure 3:**
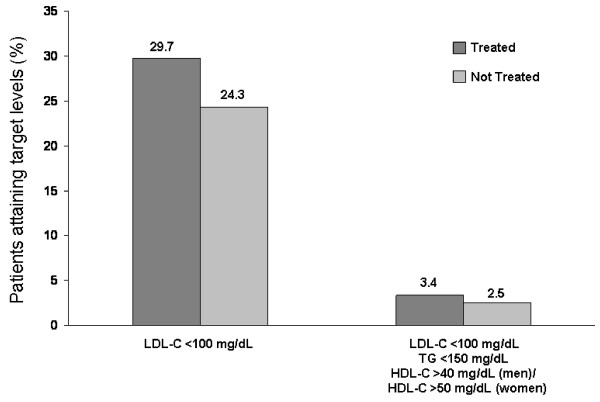
**Prevalence of target low-density lipoprotein cholesterol (LDL-C) and combined LDL-C, high-density lipoprotein cholesterol (HDL-C) + triglyceride (TG) levels for patients with type 2 diabetes mellitus either treated or not treated for dyslipidemia: National Health and Nutrition Examination Survey 1999-2000 **[27].

Data from the United States National Health and Nutritional Examination Survey (NHANES) 1999-2000 show that < 50% of 498 adults with type 2 diabetes had total cholesterol (TC) levels < 200 mg/dL despite > 50% of these patients receiving medication for dyslipidemia [4,27]. Furthermore, there was little difference in the proportion of patients with LDL-C < 100 mg/dL in the population with type 2 diabetes versus the nondiabetic population (25.3% vs 24.3%, respectively). However, significantly fewer patients with type 2 diabetes had HDL-C and TG at optimal levels compared with patients without type 2 diabetes (Figure 2) [27]. Consistent with the data from NHANES 1999-2000, data from the United Kingdom Prospective Diabetes Study (UKPDS) in 3713 newly diagnosed patients with type 2 diabetes reported that HDL-C levels were lower (by 9% [men] and 23% [women]) while TG levels were 50% higher in patients with type 2 diabetes than in nondiabetic patients; LDL-C values were similar (for males but higher in females) between those with and without type 2 diabetes [2]. As the pattern of elevated lipids was more pronounced in females, it was suggested to contribute to the greater cardiovascular risk in females compared with males [2]. Data from NHANES 1999-2000 showed that among patients with type 2 diabetes receiving treatment for dyslipidemia, control of LDL-C was only achieved in 29.7% of patients, and optimal levels of LDL-C, HDL-C, and TG were only achieved in 3.4% of patients; these data were comparable to those in patients with type 2 diabetes who were untreated for dyslipidemia (Figure 3) [27].

## Cardiovascular risk associated with dyslipidemia and treatment goals for patients with type 2 diabetes

In SHIELD (Study to Help Improve Early evaluation and management of risk factors Leading to Diabetes), a community-based population survey conducted in the United States, a multivariate analysis of self-reported data from 22,001 patients showed that dyslipidemia was independently associated with a higher likelihood of type 2 diabetes diagnosis (odds ratio, 3.95; *P <*0.0001) [28]. Moreover, it is increasingly recognized that the risk of cardiovascular events is increased in patients with type 2 diabetes and that dyslipidemia is a contributing factor with often fatal outcomes [9]. Optimal management strategies have not been fully elucidated. Although the focus has largely been on lowering LDL-C levels, the benefits associated with targeting other lipids and lipoproteins are emerging.

EUROASPIRE (EUROpean Action on Secondary Prevention through Intervention to Reduce Events) was a large survey performed to assess current clinical practice in relation to reporting and management of risk factors in the secondary prevention of coronary heart disease (CHD). Data from EUROASPIRE showed a high prevalence of modifiable risk factors, including smoking, weight, hypertension, dyslipidemia, type 2 diabetes, and lack of screening in first-degree blood relatives, among patients with CHD [29].

### LDL-C

The relationship between LDL-C and CVD risk is continuous over a broad range of LDL-C levels in patients with and without type 2 diabetes, and the National Cholesterol Education Program Adult Treatment Panel III (NCEP ATP III) recommends target lipid levels for patients with type 2 diabetes and dyslipidemia (Table 2) [30].

**Table 2:** Classification of Lipid Levels from the National Cholesterol Education Program Expert Panel [30]

Level (mg/dL)	Classification
*TC*	
< 200	Desirable
200-239	Borderline high
≥ 240	High

*LDL-C*	
< 100	Optimal
100-129	Near or above optimal
130-159	Borderline high
160-189	High
≥ 190	Very high

*VLDL-C*	
≤ 30	Normal

*HDL-C*	
< 40	Low
≥ 60	High

*TG*	
< 150	Normal
150-199	Borderline high
200-499	High
≥ 500	Very high

HDL-C, high-density lipoprotein cholesterol; LDL-C, low-density lipoprotein cholesterol; TC, total cholesterol; TG, triglyceride; VLDL-C, very-low-density lipoprotein cholesterol.

Elevated LDL-C levels are more pathogenic in those with type 2 diabetes due to the presence of small, dense LDL particles and other potentially atherogenic lipoproteins, such as VLDL and intermediate-density lipoprotein (IDL) [9,14,17,31], and data have demonstrated that lowering LDL-C levels reduces the risk for major CVD events in patients with type 2 diabetes [8]. Data from CARDS (Collaborative Atorvastatin Diabetes Study) demonstrated a clinical benefit (37% reduction in the first incidence of a major cardiovascular event; *P *= 0.001) with atorvastatin in patients (n = 2838) with type 2 diabetes whose lipid profile was not highly elevated, highlighting the importance of lipid modification for primary prevention of CVD in patients with type 2 diabetes [32]. Similarly, in the Heart Protection Study (HPS) in patients (n = 2912) with type 2 diabetes and no known coronary or other occlusive disease, simvastatin was associated with a 33% reduction (*P *= 0.0003) in coronary event rate over 5 years of treatment [33]. Importantly, the CVD benefits observed in CARDS and HPS were associated with a reduction in LDL-C, independent of baseline LDL-C level [8].

Further evidence of cardiovascular risk reduction with intensive control of LDL-C in patients with type 2 diabetes comes from ASCOT-LLA (Anglo-Scandinavian Cardiac Outcomes Trial-Lipid-Lowering Arm) [34]. In the cohort of 2532 patients with type 2 diabetes with well-controlled (average or below) BP and cholesterol levels, atorvastatin was associated with a reduction of 23% (*P *= 0.04) in total major CVD events or procedures compared with placebo; corresponding reductions in TC and LDL-C levels were 17% and 27%, respectively [34]. In addition, the Treating to New Targets (TNT) study demonstrated the benefits of reducing LDL-C to below currently recommended levels in higher-risk patients. In the 1501 patients with type 2 diabetes and CVD, intensive statin therapy provided an additional 25% reduction in major CVD events over 5 years (*P *= 0.03) [35]. In a meta-analysis of data from 14 randomized trials in 18,686 patients with diabetes (17,220 patients with type 2 diabetes), statin therapy was associated with a significant reduction in all-cause mortality (*P *= 0.02) per mmol/L reduction in LDL-C, reflecting a significant reduction in vascular mortality (*P *= 0.008). Reductions were seen regardless of prior history of vascular disease and other baseline characteristics and were similar to those seen in patients without diabetes [36]. These studies support recommendations from the American Diabetes Association (ADA) of LDL-C targets for patients with type 2 diabetes of < 100 mg/dL in low-risk patients and < 70 mg/dL in high-risk patients (Table 1), as well as reducing LDL-C by 30% to 40% in all patients aged > 40 years, and in all patients with overt CVD, regardless of baseline LDL-C status; these recommendations are also supported by the American Heart Association [8].

Although there appears to be almost overwhelming evidence for links between dyslipidemia and cardiovascular risk and between normalization of lipid levels and reduced cardiovascular risk, the data are not completely consistent. Positive data should be viewed in context with more negative data such as those seen in ASPEN (Atorvastatin Study for Prevention of coronary heart disease Endpoints in Non-insulin-dependent diabetes mellitus) [37]. ASPEN was a 4-year double-blind, randomized trial to assess the effect of atorvastatin on CVD prevention in 2410 patients with type 2 diabetes and normal LDL-C levels. In this study, mean LDL-C was significantly lowered with atorvastatin versus placebo (*P *< 0.0001); however, the composite primary endpoint (cardiovascular death, nonfatal cardiovascular events) and secondary individual endpoints were not significant [37]. Available data also suggest that increasing HDL-C levels alone does not reduce cardiovascular morbidity or mortality. In a meta-analysis of 108 randomized trials involving 299,310 patients at risk of cardiovascular events, changes in HDL-C did not correlate with cardiovascular outcomes or mortality, and the results support LDL-C reduction as the primary goal for lipid-modifying interventions [38].

### TG, HDL-C, and Non-HDL-C

In the UKPDS, a multivariate analysis found that LDL-C was the strongest independent predictor of CVD, followed by HDL-C, while modestly elevated TG levels did not predict CVD events [6], helping to justify current national guidelines advocating LDL-C < 100 mg/dL as the primary target for the management of dyslipidemia in patients with type 2 diabetes (Table 1). Serum TG levels are a surrogate for TG-rich lipoproteins (eg, VLDL-C), and non-HDL-C (LDL-C + VLDL-C or TC - HDL-C) reflects the concentration of cholesterol within lipoprotein particles considered atherogenic [8,9]. Currently, there is no evidence to demonstrate that lowering TG levels is associated with a reduction in CVD events; as a result, treatment to reduce TG levels in patients with type 2 diabetes is of secondary importance (Table 1) [9]. While HDL-C level is a strong CVD risk predictor, clinical evidence supporting the benefits of treatment aimed at increasing HDL-C levels is modest; as a result, therapy aimed at altering HDL-C in patients with type 2 diabetes is also of secondary importance (Table 1) [9]. Although not widely adopted, the ADA/American College of Cardiology (ACC) support the NCEP ATP III recommendation to use non-HDL-C as a secondary treatment target for patients with TG levels > 200 mg/dL; in these patients, the recommended non-HDL-C goal is 30 mg/dL higher than the LDL-C goal (Table 1) [9].

### ApoB

The small, dense LDL particles that accompany insulin resistance provide a better assessment of the atherogenic lipoprotein load than LDL-C level. Despite continued recommendations to reduce LDL-C in all patients with type 2 diabetes, the ADA/ACC has recognized limitations (including cost, complexity, and lack of standardized measurement techniques) in using LDL-C as a biomarker to guide therapeutic decisions for patients at high metabolic risk [9]. LDL particle concentration and apoB level appear to be more closely associated with type 2 diabetes and insulin resistance than LDL-C or non-HDL-C and may therefore be better predictors of vascular events [9,16,39,40]. Although it is possible to assess risk by measuring LDL particle concentration using nuclear magnetic resonance (NMR) [41], this technique is not widely available, relatively expensive, and of dubious accuracy [9]. However, given that there is one particle of apoB in each atherogenic lipoprotein particle (ie, LDL, IDL, and VLDL), quantification of apoB should capture the total burden of the most atherogenic particles [9,16,40], and thus serve as a valuable marker of CVD risk [14]. Although the apoB assay has been standardized and does not require a fasting sample, it is not yet widely available. Nevertheless, secondary targets of therapy have been recommended for apoB (for patients with uncomplicated type 2 diabetes [< 90 mg/dL] and for patients with type 2 diabetes and other major risk factors [< 80 mg/dL]) [9].

### ApoA-I

Data from epidemiological, observational, and interventional studies also suggest an association between apolipoprotein A-I (apoA-I) and cardiovascular risk, indicating that assessment of apoA-I in addition to apoB may provide more accurate prediction of high cardiovascular risk than conventional lipid parameters [42]. The ratio of apoB to apoA-I has been shown to correlate with cardiovascular risk in children [43].

## Improving dyslipidemia in patients with type 2 diabetes

Achievement of recommended lipid and lipoprotein targets usually requires pharmacological therapy in addition to lifestyle modifications (a low-fat/cholesterol diet and physical activity). Although aggressive LDL-C control is recommended [10], evidence-based strategies are needed to monitor the benefits and risks of pharmacotherapy aimed at surrogate markers [44] and to evaluate whether lower targets do indeed achieve better cardiovascular outcomes [45].

### Lipid-lowering agents

#### Statins

Statin therapy is recommended as the initial pharmacological treatment for lowering LDL-C levels in patients with type 2 diabetes who either have overt CVD or are over 40 years old and have increased CVD risk [8,10]; however, even with adequate LDL-C lowering via statin therapy, CVD risk remains high in many patients [8,9]. The beneficial effects of statin treatment are thought to be mediated predominantly via lowering of LDL-C levels, although effects on HDL-C and other lipoproteins may also play a role [9]. Statin treatment lowers non-HDL-C more than apoB [40], and reaching the apoB target usually requires more intensive therapy than that required to achieve the non-HDL-C goal [46,47]. Common adverse events associated with statin use include gastrointestinal upset and muscle aches, although dose-related hepatoxicity and myotoxicity are the most clinically significant adverse events [48]. Caution is recommended in patients with severe renal impairment (creatinine clearance < 30 mL/min). Studies have shown that high-dose statin therapy is effective in achieving LDL-C goals and associated with favorable effects on lipoprotein subfractions in patients with type 2 diabetes, which may translate into clinical benefits in terms of anti-atherogenic potential and a subsequent reduction in the risk of adverse cardiovascular outcomes [49,50].

#### Other lipid-lowering therapies

Niacin has been used to treat dyslipidemia in patients with type 2 diabetes for over half a century [51]. Although niacin is the most effective agent for raising HDL-C levels, high doses can worsen hyperglycemia [10]. Additional adverse events associated with niacin include flushing, itching, nausea, gastrointestinal upset, hypotension, and tachycardia [48,51]. It has been suggested that combination lipid-lowering therapy (eg, a statin with a fibrate or niacin) may be necessary for patients with diabetic dyslipidemia to achieve optimal lipid levels; however, to date, such strategies have not been adequately evaluated for their long-term effect on CVD risk reduction or safety compared with lipid-lowering monotherapy [8-10]. Furthermore, the risk of myopathy is thought to be greater when niacin is used with a statin [51]. Niacin plus laropiprant - a prostaglandin D_2 _receptor antagonist and antiflushing agent - has been used successfully to improve the lipid profile with reduced niacin-associated flushing in patients with type 2 diabetes [52]. In 2 large randomized studies in patients with primary hypercholesterolemia or mixed dyslipidemia, the combination of niacin, laropiprant, and simvastatin significantly improved lipid parameters with a similar tolerability profile versus niacin/laropiprant alone, but with an increase in flushing and other niacin-related adverse effects versus statin alone [53,54].

Ezetimibe, a selective cholesterol absorption inhibitor, is an effective lipid-lowering agent when used as monotherapy and is useful in patients who are unable to tolerate statin therapy [51]. Ezetimibe can also be used in combination with statin therapy for greater lipid-lowering efficacy. Ezetimibe plus atorvastatin, for example, can provide LDL-C lowering equivalent to that achieved with high-dose atorvastatin, but with better tolerability in some patients, and may be a useful adjunctive therapy in patients with type 2 diabetes who have demonstrated an inadequate response to statin treatment [55].

Fibrates are useful for lowering TG and non-HDL-C levels and increasing HDL-C, yet results from trials in patients with type 2 diabetes have been controversial [56]. In the FIELD (Fenofibrate Intervention and Event Lowering in Diabetes) study in 9795 patients with type 2 diabetes, fenofibrate did not significantly affect the primary endpoint, coronary event rate, relative to placebo (11% reduction) [57]. Nevertheless, FIELD did show that combination therapy with a statin and fenofibrate is safe. Recent results from the ACCORD (Action to Control Cardiovascular Risk in Diabetes) study provided further insight into whether the combination of a statin and a fibrate is safe and provides CVD benefits beyond statin therapy alone. In this study in 5518 patients with type 2 diabetes, there was no difference between combination therapy with a statin and fibrate compared with statin therapy alone with respect to the primary outcome (nonfatal myocardial infarction, nonfatal stroke, or death from cardiovascular causes) [58]. Common adverse events associated with fibrates include gastrointestinal disturbance, rash, headache, pancreatitis, myalgia, and myotoxicity (in rare instances - and possibly more likely with gemfibrozil than with fenofibrate [59]). Adjuvant fibrate therapy is not recommended in patients with severe renal dysfunction, severe hepatic dysfunction, and preexisting gall bladder disease [48].

### Glucose-lowering agents

In addition to their glucose-lowering properties, antidiabetes agents that directly improve insulin resistance may have effects on lipid levels, especially TG levels. Although there may be no effect on HDL-C levels, these agents may instead alter the ratio of lipoproteins in HDL towards more anti-atherogenic HDL particles [48]. For example, metformin has been shown to reduce LDL-C, TC, and TG levels and increase HDL-C levels [60]. Similarly, pioglitazone has been shown to reduce TG levels and increase HDL-C levels [61]. In contrast, rosiglitazone has been shown to increase LDL-C, TC, and HDL-C levels, although this thiazolidinedione does not affect TG levels [62]. A prospective study evaluating the effects of 4 months of treatment with pioglitazone or rosiglitazone in addition to statin therapy in 127 patients with type 2 diabetes showed that, despite similar findings regarding HbA_1c _and weight gain (no changes from baseline in HbA_1c _and similar significant weight gain in both groups at 4 months), pioglitazone, but not rosiglitazone, was associated with significant improvements in the lipid profile (*P *< 0.01) [63]. Interestingly, both thiazolidinediones have been shown to increase LDL particle size and decrease LDL oxidation -- conditions that impair atherosclerosis (pioglitazone more so than rosiglitazone) [64]. Pioglitazone has also been reported to improve HDL-C and TG parameters when used as an add-on therapy in patients with type 2 diabetes who are already receiving metformin or sulfonylurea therapy [65,66] and is more effective in improving lipid and apolipoprotein levels than rosiglitazone plus sulfonylurea, in addition to ongoing statin therapy [67]. Although the use of sulfonylurea monotherapy has not been associated with significant changes in the lipid profile, the addition of acarbose to sulfonylurea therapy not only improves glycemic control, but also provides improvements in lipid parameters, particularly TG levels [68].

### Dual lipid- and glucose-lowering agents

Accumulating evidence indicates that lipid and glucose homeostasis are interrelated [69]. Both are affected by bile acid-activated signaling pathways in the liver [70]. Indeed, bile acids have an established role in dietary lipid absorption and cholesterol metabolism, and are also signaling molecules that affect systemic endocrine functions through multiple signaling pathways [71]. Agents that modulate bile acids may potentially affect both cholesterol and glucose metabolism, and hence dyslipidemia and hyperglycemia, in patients with type 2 diabetes [69,71,72].

Bile acid sequestrants are established agents for LDL-C lowering. The bile acid sequestrant colesevelam lowers LDL-C when used as monotherapy (up to 18%) [73] and can result in reductions of up to 48% when used in combination with a statin in patients with mild-to-moderate hypercholesterolemia [74]. In addition, colesevelam is the only lipid-lowering agent approved to improve glucose levels in patients with type 2 diabetes [75]. A pilot study, GLOWS (Glucose-Lowering effect of WelChol Study), showed that colesevelam significantly reduced LDL-C levels (11.7%) and HbA_1c _(0.5%) compared with placebo when added to existing metformin- and/or sulfonylurea-based therapy in patients with type 2 diabetes [76]. In subsequent trials, the addition of colesevelam to stable metformin-, sulfonylurea-, or insulin-based therapy resulted in additional reductions in LDL-C (12.8% to 16.7%) and HbA_1c _(0.50% to 0.54%) (*P <*0.001 vs placebo for all) [77-79]. Consistent with a significant reduction in apoB (11.8%) in GLOWS [76], improvements in atherogenic lipoprotein subclasses measured by NMR were also reported with colesevelam. The most significant effect with colesevelam was seen on LDL particles (15.5% reduction vs placebo; *P *= 0.006), primarily due to an improvement in the number of small LDL particles (*P *= 0.054 vs placebo) [80]. Notably, the reduction in LDL particles was the only lipid parameter correlated with improvement in HbA_1c _[80].

The use of bile acid sequestrants in patients with type 2 diabetes has been a concern due to an association with increased fasting TG levels. There was a nonsignificant increase in TGs and in TG-containing VLDL particles in association with colesevelam in GLOWS; however, there was a significant increase in TG levels in studies when colesevelam was added to sulfonylurea-based therapy (17.7% vs placebo; *P *< 0.001) and insulin-based therapy (21.5% vs placebo; *P *< 0.001), though not metformin-based therapy (4.7% vs placebo; *P *= 0.22) [76-79]. Adverse events associated with colesevelam include gastrointestinal disturbances such as constipation, dyspepsia, and nausea [75].

The exact mechanism by which a bile acid sequestrant may improve glycemic control in patients with type 2 diabetes is not fully understood, but may involve activation of the nuclear receptors farnesoid X receptor (FXR) in the liver and intestine and TGR5 in the intestine [72,81]. FXR is a bile acid-activated nuclear receptor involved in the metabolism of cholesterol and glucose [82,83]. Intestinal activation of FXR modulates multiple downstream effects including those that affect glucose and lipid metabolism through regulatory activity at other sites including the liver X receptor, a potential glucose sensor [70]. *In vitro*, TGR5 has been shown to improve expression of the incretin hormone glucagon-like peptide-1 (GLP-1), which improves insulin secretion. Both colestimide and colesevelam have been shown to increase GLP-1 in patients with type 2 diabetes and hypercholesterolemia [84,85]. Further studies are needed to elucidate the mechanism(s) for bile acid sequestrant-mediated regulation of glucose control.

### Antihypertensive agents

Some antihypertensive agents are known to be lipid neutral (angiotensin-converting enzyme inhibitors, calcium-channel blockers, and angiotensin II receptor blockers) or lipid friendly (α-blockers) [86]. In patients with type 2 diabetes and dyslipidemia, a lipid-neutral/friendly antihypertensive would be expected to be associated with greater clinical benefits than a lipid-hostile antihypertensive such as a β-blocker or thiazide diuretic [87].

## Dyslipidemia and renal function

In addition to being associated with an increased risk of CVD, dyslipidemia is also associated with an increased risk of renal dysfunction (increased serum creatinine elevation and decline in glomerular filtration rate) in patients with type 2 diabetes [88-90]. Chronic renal dysfunction can increase the risk of CVD. An analysis of cardiac function in patients with type 2 diabetes and end-stage renal disease showed that significantly more patients with type 2 diabetes than without had impaired cardiac function as measured by left ventricular hypertrophy (50% vs 38%, respectively; *P *= 0.04), ischemic heart disease (32% vs 18%; *P *= 0.003), and cardiac failure (48% vs 24%; *P <*0.0001) [91]. Therefore, lowering lipid levels in patients with type 2 diabetes may improve both cardiovascular risk and renal function. *Post-hoc *analyses have shown that statin therapy is associated with improved renal function in patients with diabetes and those with chronic renal insufficiency and CVD [33,92]. In the MRC/BHF Heart Protection Study, simvastatin improved renal function in patients with type 2 diabetes [33]. However, it remains unclear whether the beneficial renal effects of statins are a direct result of reduced serum cholesterol levels or an as yet undefined pleiotropic effect.

## Conclusions

Type 2 diabetes is associated with a characteristic atherogenic lipid pattern of elevated serum TGs, low serum HDL-C levels, and a preponderance of small, dense LDL particles. Disturbance of lipid metabolism linked to insulin resistance may be the primary event in the development of type 2 diabetes. The majority of adults in the United States with type 2 diabetes do not have optimal lipid profiles based on national guidelines. In order to reduce the risk of CVD in patients with type 2 diabetes, physicians must initiate early and effective lipid-lowering therapy. Although the first priority of treatment is to lower LDL-C in patients with type 2 diabetes, the atherogenic pattern of dyslipidemia associated with type 2 diabetes may require an advanced treatment approach that ultimately aims for full normalization of the lipid profile to decrease cardiovascular risk. Data from combined prevalence studies suggest that potentially all patients with type 2 diabetes may have an abnormal lipid profile. Despite aggressive lipid-lowering therapy, many patients with type 2 diabetes do not achieve the recommended lipid levels to reduce their CVD risk sufficiently. Adjuvant use of a bile acid sequestrant such as colesevelam, having the dual effect of improving both glycemic control and atherogenic profile in patients with type 2 diabetes, may help improve the overall management of type 2 diabetes in some patients.

## Abbreviations

ACC: American College of Cardiology; ACCORD: Action to Control Cardiovascular Risk in Diabetes; ADA: American Diabetes Association; Apo: apolipoprotein; ASCOT-LLA: Anglo-Scandinavian Cardiac Outcomes Trial-Lipid-Lowering Arm; ASPEN: Atorvastatin Study for Prevention of coronary heart disease Endpoints in Non-insulin-dependent diabetes mellitus; BP: blood pressure; CARDS: Collaborative Atorvastatin Diabetes Study; CETP: cholesterol ester transfer protein; CHD: coronary heart disease; CVD: cardiovascular disease; EUROASPIRE: EUROpean Action on Secondary Prevention through Intervention to Reduce Events; FIELD: Fenofibrate Intervention and Event Lowering in Diabetes; FXR: farnesoid X receptor; GLOWS: Glucose-Lowering effect of WelChol Study; GLP-1: glucagon-like peptide-1; HDL-C: high-density lipoprotein cholesterol; HPS: Heart Protection Study; IDL: intermediate-density lipoprotein; LDL-C: low-density lipoprotein cholesterol; NCEP ATP III: National Cholesterol Education Program Adult Treatment Panel III; NEFA: nonesterified (free) fatty acid; NHANES: United States National Health and Nutritional Examination Survey; NMR: nuclear magnetic resonance; Non-HDL-C: non-high-density lipoprotein cholesterol; RAAS: renin-angiotensin-aldosterone system; SHIELD: Study to Help Improve Early evaluation and management of risk factors Leading to Diabetes; TC: total cholesterol; TG: triglyceride; TNT: Treating to New Targets; UKPDS: United Kingdom Prospective Diabetes Study; VLDL: very-low-density lipoprotein cholesterol.

## Competing interests

KV has received honoraria from Daiichi Sankyo, Inc. as a member of their advisory board.

## Authors' contributions

KV has read and approved the final manuscript.
